# Supplementary material for: From Soil to Grape and Wine: Geographical Variations in Elemental Profiles in Different Chinese Regions
Source: Foods. 2021 Dec 15;10(12):3108. doi: 10.3390/foods10123108 (PMC8701803; doi:10.3390/foods10123108)
Supplement: Supplementary file 1 [file foods-10-03108-s001.zip › foods-1403466-supplementary.pdf]

Supplemental Data:

Supplementary Table S1 Bioaccumulation factors of elements in system soil-grape.

BAF	YL	BLY	HY	XX	MQ	WH
Na	<0.01b	<0.01b	<0.01b	<0.01a	<0.01a	<0.01b
Mg	0.03±0.01ab	0.02±0.01b	0.04±0.01a	0.04±0.01a	0.04±0.01a	0.04±0.01a
K	0.10±0.01b	0.13±0.01a	0.13±0.01a	0.11±0.02ab	0.10±0.02b	0.11±0.01ab
Ca	0.01±0.01a	0.01±0.003a	0.01±0.002a	0.01±0.002a	0.01±0.003a	0.01±0.004a
Al	<0.01b	<0.01b	<0.01a	<0.01b	<0.01b	<0.01b
Fe	<0.01c	<0.01c	<0.01b	<0.01bc	<0.01a	<0.01a
Mn	<0.01b	<0.01c	<0.01b	<0.01a	<0.01b	<0.01b
Cu	0.09±0.01abc	0.06±0.01c	0.13±0.13abc	0.18±0.09ab	0.20±0.10a	0.14±0.02abc
Zn	0.02±0.003b	0.02±0.01ab	0.02±0.01ab	0.03±0.01a	0.03±0.01a	0.03±0.01a
Rb	0.23±0.10c	1.21±0.29a	0.47±0.20b	0.10±0.09c	0.07±0.03c	0.02±0.01c
Sr	<0.01c	<0.01c	<0.01c	0.03±0.007a	<0.01c	0.02±0.005b
Li	<0.01d	<0.01e	<0.01de	<0.01c	<0.01b	<0.01a
Cd	<0.01a	<0.01a	<0.01b	<0.01d	<0.01bc	<0.01cd
Cs	<0.01cd	0.01±0.004a	<0.01b	<0.01bc	<0.01cd	<0.01d
Ba	0.05±0.03a	<0.01c	0.02±0.002b	0.05±0.02a	0.05±0.02a	0.04±0.01ab

Note: Different letters in the same row indicate statistically significantly different ($P < 0.05$).

Supplementary Table S2 Pearson's correlation analysis of BCF values between the elements.

	Na	Mg	Al	K	Ca	Mn	Fe	Cu	Zn	Rb	Sr	Li	Cd	Cs	Ba
Na	1														
Mg	.232	1													
Al	-.225	.256	1												
K	-.307	-.037	.342	1											
Ca	-.135	.593**	.015	-.061	1										
Mn	.385*	.571**	.091	-.069	.250	1									
Fe	.315	.539**	.075	-.109	.284	.038	1								
Cu	.418*	.371*	.107	-.223	.082	.255	.514**	1							
Zn	.344	.533**	-.188	.063	.420*	.212	.585**	.357	1						
Rb	-.480**	-.467**	.082	.599**	-.021	-.511**	-.485**	-.451*	-.145	1					
Sr	.435*	.370*	-.255	-.053	.079	.784**	-.043	.264	.308	-.446*	1				
Li	.061	.455*	-.206	-.157	.163	.097	.593**	.277	.347	-.579**	.339	1			
Cd	-.433*	-.574**	-.113	-.129	.030	-.450*	-.400*	-.377*	-.312	.440*	-.603**	-.590**	1		
Cs	-.298	-.374*	-.003	.493**	.025	-.505**	-.217	-.296	.035	.910**	-.479**	-.503**	.353	1	
Ba	.312	.551**	-.071	-.503**	.323	.506**	.283	.374*	.096	-.657**	.335	.279	-.264	-.618**	1

Note: * Correlation is significant at the 0.05 level.

** Correlation is significant at the 0.01 level.

Supplementary Table S3 Transfer factors of elements in system wine-grape.

TF	YL	BLY	HY	XX	MQ	WH
Na	1.89±0.16abc	2.19±0.28ab	1.53±0.19c	2.43±0.68a	1.57±0.18bc	2.04±0.19abc
Mg	1.64±0.58a	1.46±0.19a	1.59±0.28a	1.43±0.16a	1.71±0.36a	1.4±0.21a
K	1.16±0.07b	0.98±0.05c	0.97±0.07c	1.33±0.1a	1.19±0.1b	0.86±0.06c
Ca	0.58±0.23a	0.63±0.13a	0.53±0.18a	0.35±0.1a	0.54±0.19a	0.4±0.1a
Al	0.29±0.03a	0.35±0.04a	0.3±0.09a	0.46±0.17a	0.33±0.01a	0.42±0.22a
Fe	0.3±0.1a	0.23±0.11a	0.3±0.06a	0.35±0.07a	0.27±0.04a	0.32±0.05a
Mn	0.54±0.12a	0.69±0.13a	0.58±0.13a	0.55±0.1a	0.8±0.15a	0.63±0.17a
Cu	0.09±0.01b	0.19±0.03a	0.12±0.06b	0.09±0.04b	0.09±0.02b	0.15±0.02ab
Zn	1.15±0.21a	0.54±0.21b	1.04±0.3a	1.18±0.26a	0.96±0.14a	0.85±0.18ab
Rb	0.83±0.14a	0.64±0.03a	0.6±0.05a	0.83±0.16a	0.71±0.34a	0.76±0.24a
Sr	0.45±0.08bc	0.7±0.07a	0.49±0.09b	0.29±0.05c	0.55±0.16ab	0.42±0.09bc
Li	1.12±0.13b	0.74±0.08c	0.95±0.05bc	1.57±0.27a	1.58±0.32a	1.08±0.1b
Cd	0.22±0.03b	0.66±0.06a	0.74±0.31a	0.83±0.2a	0.62±0.16a	0.64±0.09a
Cs	1.23±0.12a	0.89±0.04a	0.91±0.04a	0.88±0.26a	0.91±0.35a	1.1±0.48a
Ba	0.44±0.13ab	0.63±0.13a	0.49±0.1ab	0.3±0.08b	0.44±0.16ab	0.43±0.04ab

Note: Different letters in the same row indicate statistically significantly different ($P < 0.05$).
